# Characteristics of alcohol care teams in England: results of the ProACTIVE National Survey

**DOI:** 10.1093/alcalc/agaf082

**Published:** 2025-12-30

**Authors:** Philippa Case, Madeleine Wilkinson, Georgia Foote, Nicola Kalk, Colin Angus, Krysia Canvin, Judith Cohen, Colin Drummond, Steven Masson, Amy O’Donnell, Seilin Uhm, Julia Sinclair, Thomas Phillips

**Affiliations:** Centre for Addiction and Mental Health Research, Faculty of Health Sciences, University of Hull, Cottingham Road, Hull, HU6 7RX, United Kingdom; Centre for Addiction and Mental Health Research, Faculty of Health Sciences, University of Hull, Cottingham Road, Hull, HU6 7RX, United Kingdom; Faculty of Medicine, University of Southampton, 12 University Road, Southampton, SO17 1BJ, United Kingdom; Kings College Hospital NHS Foundation Trust, Kings College Hospital, Denmark Hill, London, SE5 9RS, United Kingdom; National Addiction Centre, Institute of Psychiatry, Psychology & Neuroscience, Kings College London, Addiction Sciences Building, 4 Windsor Walk, London, SE5 8AF, United Kingdom; School of Medicine and Population Health, University of Sheffield, Beech Hill Road, Sheffield, S10 2RX, United Kingdom; School of Medicine, Keele University, University Road, Staffordshire, ST4 6QG, United Kingdom; Centre for Addiction and Mental Health Research, Faculty of Health Sciences, University of Hull, Cottingham Road, Hull, HU6 7RX, United Kingdom; Hull Health Trials Unit, Hull York Medical School, University of Hull, Cottingham Road, Hull, HU6 7RX, United Kingdom; National Addiction Centre, Institute of Psychiatry, Psychology & Neuroscience, Kings College London, Addiction Sciences Building, 4 Windsor Walk, London, SE5 8AF, United Kingdom; The Newcastle upon Tyne Hospitals NHS Foundation Trust, Freeman Hospital, Freeman Road, High Neaton, Newcastle upon Tyne, NE7 7DN, United Kingdom; Faculty of Medical Sciences, The Medical School, Newcastle University, Framlington Place, Newcastle upon Tyne, NE2 4HH, United Kingdom; Population Health Sciences Institute, Faculty of Medical Sciences, Newcastle University, Richardson Road, Newcastle upon Tyne, NE2 4AX, United Kingdom; Faculty of Environmental and Life Sciences, University of Southampton, University Road, Southampton, SO17 1BJ, United Kingdom; Faculty of Medicine, University of Southampton, 12 University Road, Southampton, SO17 1BJ, United Kingdom; Centre for Addiction and Mental Health Research, Faculty of Health Sciences, University of Hull, Cottingham Road, Hull, HU6 7RX, United Kingdom

**Keywords:** alcohol use disorder, hospital, alcohol care team, consultation liaison service, care components

## Abstract

**Aims:**

This study aimed to identify (i) the number of alcohol care teams (ACTs) in England, (ii) the characteristics of patients supported by ACTs, and (iii) the service structure and care components offered by ACTs.

**Methods:**

All acute hospitals (i.e. those providing short-term high-dependency medical care) in England were approached to complete a survey of alcohol care provision. Surveys were completed through researcher-guided interviews by staff familiar with the hospital’s alcohol provision. It featured questions on service structure, patient characteristics, service functions, and policies. Data collection took place between May and October 2023.

**Results:**

Of 170 hospitals approached, 122 completed a survey and 80 reported having an ACT. Most ACT patients were male (mean 64.1%; 95% confidence interval (CI) 61.8–66.4), white (mean 79.2%; 95% CI 75.1–83.4), aged 45–54 (mean 27.8%; 95% CI 25.0–30.5), and experiencing severe alcohol dependence (mean 66.2%; 95% CI 36.8–95.7). Most services had a clinical lead but only 58% funded this role. Fifty-nine percent of services operated 7 days per week. Most services reported identification and brief advice, though it was rarely systematized. Nearly all supported medically assisted alcohol withdrawal, though a quarter of patients did not complete medically assisted alcohol withdrawal before discharge.

**Conclusions:**

ACT numbers increased significantly between 2019 and 2024. They offer a clinical service to highly vulnerable and complex patients. There is significant variation in ACT operational models, training, and leadership which will impact the effectiveness of identification strategies and management of patients with comorbid alcohol use disorder within acute medical settings.

## Teaser

**Short summary** Alcohol care teams (ACTs) offer a clinical service to highly vulnerable and complex patients. This national survey of ACTs in England found significant variation in ACT operational models, training, and leadership which will impact the effectiveness of identification strategies and management of patients with comorbid AUD within acute medical settings.

## Introduction

Alcohol consumption is linked to more than 200 medical conditions (WHO 2024). Chronic alcohol disorders including harmful drinking, alcohol use disorder (AUD), and alcohol-related liver disease (ArLD) have a disproportionate impact on health services (Phillips et al. 2019). In 2023/24, there were 1 018 986 alcohol-related hospital admissions in England (OHID 2025a), and it is estimated that one in 5 hospital patients in the UK drink alcohol at harmful levels and one in 10 are alcohol-dependent (Roberts et al. 2019). Hospital readmissions are common in this population, with data from England indicating a 30-day readmission rate of 20.2% (95% CI 19.4–20.8) for those admitted with an alcohol-related condition, particularly for those with coexisting physical and mental health problems, or who discharge against medical advice (Phillips et al. 2025).

UK Government strategies have supported the development of hospital-based alcohol care initiatives in response to rising admissions, resulting in the expansion of these services from 2008 (UK Government 2004, 2007, 2012), with the aim of reducing alcohol-related admissions by improving quality of care and facilitating engagement with community services (NHS England (NHSE) 2019a). A 2013 survey of 191 acute hospitals in England identified a minimum of 73% of hospitals reported some level of specialist alcohol care provision (Public Health England (PHE) 2014). These services fell into three broad operational models: (i) specialist consultation liaison teams [alcohol care teams (ACTs)]; (ii) specialist in-reach staff provided by community addiction services; and (iii) community-based high impact service-user teams (e.g. alcohol assertive outreach teams).

In 2019, the UK Government’s NHS Long Term Plan (NHSE 2019b) committed a further £26 m over 4 years (until mid-2024) to ‘optimize’ ACTs through the development of existing services and the establishment of new teams in the 25% of hospitals in areas of greatest need in England. In this context, ‘optimization’ involved delivering a 7-day service providing both patient and system level interventions to improve the identification and management of alcohol-dependent patients. Detailed service descriptors, outlining the core components of ACTs (NHSE 2019c), were developed based on a decade of evolving clinical experience and policies (Moriarty 2011, PHE 2014, NHSE 2019a, NHSE 2019c) and are described in Box 1. The rationale for these components, as well as a 7-day per week service, coordinated alcohol policies, alcohol assertive outreach services (AAOT) for frequent attenders, and quality metrics and monitoring is described elsewhere (Moriarty 2020).

Box 1. Service descriptor with core components of ACTs (NHSE 2019c)

A multi-disciplinary ACT should provide packages of care that include:

- Case identification and brief advice (IBA)
- Comprehensive alcohol assessment
- Specialist nursing and medical care planning
- Management of medically assisted alcohol withdrawal (MAAW)
- Provision of psychosocial interventions
- Planning safe discharge, including referral to community services
- Clinical leadership by a senior clinician with dedicated time for the team
- Provision of trust-wide education and training in relation to alcohol

To date no information on the characteristics of patients supported by ACTs, nor any systematic data on the geographical spread, structure and components of care, including fidelity to the core service descriptor, have been published.

The aims of this study were to identify (a) the number of ACTs in England, (b) the characteristics of patients supported by ACTs, and (c) the service structure and components of care offered by ACTs. The study is part of a wider programme of research (ProACTIVE); a multidisciplinary, integrated mixed methods programme designed to evaluate the impact, value and effectiveness of ACTs at the patient (micro), health system (meso) and policy (macro) levels (https://fundingawards.nihr.ac.uk/award/NIHR152084).

## Materials and methods

### Procedure

All acute hospitals (i.e. those providing short-term high-dependency medical care for acute illnesses or injuries) in England were approached to complete a survey of alcohol care provision. Surveys were completed by staff members who were sufficiently familiar with the hospital’s alcohol provision to be able to answer questions about its structure, care components, and patient characteristics. Respondents signed an electronic consent form following receipt of an information sheet and were encouraged to attend an online researcher-guided interview to complete the survey. Five respondents opted to complete the survey independently and data for these five sites were checked by a researcher, and respondents were followed up with any data queries. All data collection took place between May and October 2023. Ethical approval for this study was granted by the NHS Health Research Authority (HRA) and Health and Care Research Wales [REC reference: 23/HRA/0311; IRAS project ID: 322240].

### Materials

The survey was developed in consultation with clinicians experienced in delivering hospital-based alcohol services, and academics with experience of conducting research in this setting. Questions from the previous survey conducted by Public Health England were adapted with the aim of capturing fidelity to the core service descriptor provided by NHS England (NHSE 2019c). The online survey comprised questions about the hospital [e.g. size, presence of a consultant-led emergency department (ED)] and whether there was current ‘access to dedicated alcohol care available to patients’ who (1) attend ED and (2) are admitted to hospital. If respondents answered ‘no’ to both above, they were asked about the presence of hospital policies relating to alcohol care only.

For hospitals reporting alcohol care, additional questions were asked on (i) the structure of the hospital-based alcohol service (e.g. operating hours, leadership, staffing); (ii) referrals (e.g. number, source, outcome); (iii) characteristics of patients seen (e.g. gender, ethnicity, age, severity of AUD); (iv) service activities and functions (e.g. alcohol identification and brief advice (IBA), management of medically assisted alcohol withdrawal); and (v) the existence of wider hospital policies and pathways for alcohol-related care. For severity of AUD, respondents were asked to estimate what proportion of patients assessed by their teams were: hazardous drinkers (defined as: ‘Drinking above 14 units per week or AUDIT Score 8-15’); harmful drinkers (defined as: ‘Experiencing social, psychological or physical problems due to drinking but NOT dependence, or AUDIT Score 16-19’); experiencing moderate dependence (defined as: ‘Experiencing significant level of tolerance, alcohol withdrawal, impaired control, typically may be drinking 15-30 units per day’); or experiencing severe dependence (defined as: ‘Experiencing severe alcohol withdrawal, high tolerance, typically drinking 30 units or more per day and may have experienced withdrawal seizures or delirium tremens’). The full survey tool is available on request.

All data were self-reported. Questions were framed in terms of ‘a typical month’ in the last 12 months, and respondents were asked about the proportion of assessments (e.g. male vs female) rather than the number of assessments, for data to be comparable across different sized sites. All data were analysed using Stata v18.0.

Respondents reporting any hospital-based alcohol care were asked to categorize their service into ACT, In-Reach service, alcohol assertive outreach teams (AAOT), rapid assessment, interface and discharge (RAID), or ‘Minimal’ models (definitions of these service models derived from existing evidence and adapted by the ProACTIVE Consortium of researchers, public contributors, and clinicians for the purpose of the survey, are contained in Box 2). Services could also select multiple component models. For the purpose of this analysis, any multicomponent service that included ACT was considered an ACT model. Respondents were asked when their service became clinically active and were grouped between those clinically active pre- and post the additional funding through the NHS Long Term Plan (NHSE 2019b).

Box 2. Definitions of hospital-based alcohol services

**Multi-disciplinary ACTs:** ‘a funded service based within the hospital and/or within a specific department (e.g. ED, Gastro/Hepatology), with 2 or more staff which supports the screening, assessment and treatment of alcohol disorders, as well as training across the hospital. This service should be available on 5 or more days per week’.

**In-Reach** alcohol services: ‘a team of two or more staff based outside the hospital who usually provide peripatetic support from the specialist community alcohol services who regularly attend to provide interventions (e.g. Screening, assessment and training)’.

**Alcohol Assertive Outreach Teams (AAOT)**: ‘a team of two or more staff based outside the hospital who may be integrated intensive support in the community and would regularly attend admitted patients to provide interventions (e.g. Screening, assessment and training) and support pathways to community services’.

**Rapid Assessment Interface and Discharge (RAID)**: ‘a model of liaison mental health service that incorporates an alcohol care pathway’.

The commissioning arrangements and needs of the hospital and community populations meant that hospitals with high levels of need incorporated a system of care with one or more of the above services, whereas several hospitals described a:

**Minimal Service**, which was defined as ‘a single member of staff or occasional visiting professional’.

## Results

**Figure 1:**
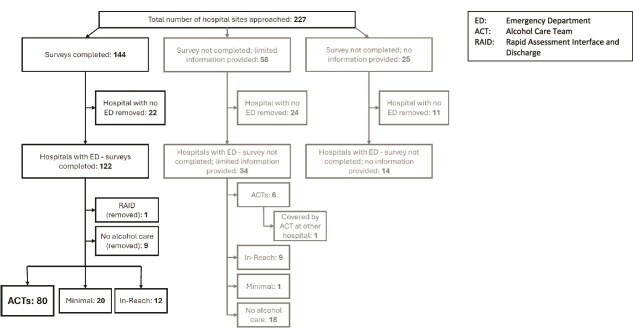
Recruitment diagram

Of the 227 hospitals that were approached to participate, respondents representing144 hospitals completed a survey, 58 did not complete a survey but provided limited information, and 25 did not participate and gave no information. Due to wide variation in the service provision of smaller hospitals without EDs, we restricted the analysis to hospitals with an ED. One hundred and seventy hospitals with a 24-h ED were approached, of which respondents representing 122 participated in the survey, 34 did not complete a survey but provided limited information on their service, and 14 did not participate and gave no information (Fig. 1 and Table 1).

**Table 1:** Regional response rate and distribution of alcohol-based care (hospitals with emergency department).

					Of the services that completed the survey and reported alcohol care provision (*n* = 113)			
Region	No. of hospitals approached	Survey respondents reporting some alcohol care provision	No. of reporting no alcohol care^a^	No. of completed survey or reported no alcohol care (% hospitals approached: response rate)	ACT (self-described) *n* (% hospitals with alcohol care)	Minimal *n* (%)	In-reach *n* (%)	Rapid assessment *n* (%)
East of England	19	9	4	13 (68)	4 (44)	2 (22)	3 (33)	0
London	26	20	3	23 (88)	14 (70)	5 (25)	1 (5)	0
Midlands	26	20	1	21 (81)	12 (60)	3 (15)	4 (20)	1 (5)
North East and Yorkshire	30	19	9	28 (93)	15 (79)	2 (11)	2 (11)	0
North West	25	14	1	15 (60)	14 (100)	0	0	0
South East	27	17	7	24 (89)	11 (65)	4 (23)	2 (12)	0
South West	17	14	2	16 (94)	10 (71)	4 (29)	0	0
**Total**	**170**	**113**	**27**	**140 (82)**	**80 (71)**	**20 (18)**	**12 (11)**	**1 (<1)**

^a^Including nine hospitals which responded to the survey and reported no alcohol care and a further 18 which reported no alcohol care and did not complete the survey.

ACT, alcohol care team.

The figures in bold represent totals for each column.

### Aim 1: To identify the number of alcohol care teams in England

Of the 122 hospitals with EDs which completed a survey, 9 (8%) reported no hospital-based alcohol care, while 113 hospitals reported some alcohol care provision, including 80 (71%) ACTs, 20 (18%) minimal services, 12 (11%) in-reach services, and 1 (<1%) RAID service. Just under half (44%) of all ACTs surveyed reported becoming clinically active in, or after, 2019 following the introduction of the NHS Long Term Plan, with the establishment of ACTs in the largest hospitals being prioritized (69%) compared to the introduction of ACTs in medium (29%) or smaller (44%) size hospitals. ACTs were primarily in the geographical areas of highest need.

The remainder of the results will focus on those hospitals that completed the survey and reported having an ACT. Due to the likely influence of hospital size on some of the structural components of care (e.g. staffing numbers), results are presented for all ACTs (*n* = 80) and by size of hospital (*n* = 77).

### Aim 2: Identify the characteristics of patients supported by alcohol care teams in England

#### Demographic and clinical characteristics (see Table 2)

The mean number of patients assessed per month by ACTs was 138 (95% confidence interval (CI) 117–159). The lowest number of assessments was seen in the smallest hospitals, and the highest number was seen in the medium-sized hospitals (see Table 2). Most patients seen by ACTs were male, from white backgrounds and aged 45 to 54 (Table 2).

**Table 2:** Characteristics of ACT patients.

			Hospital size (based on no. of discharges per month) (*n* = 77)		
	Total *n* full sample (by hospital size)^a^	All ACTs mean % (95% CI)	Small (500–1999) mean % (95% CI)	Medium (2000–2999) mean % (95% CI)	Large (3000+) mean % (95% CI)
**Gender**	79 (77)				
Male		64.1 (61.8–66.4)	64.0 (60.2–67.7)	65.2 (62.1–68.4)	63.4 (59.3–67.5)
Female		34.6 (32.8–36.4)	33.7 (31.0–36.4)	34.8 (31.6–37.9)	36.5 (32.4–40.6)
Not specified		0.01 (−0.1 to 0.03)	0	0	0.08 (−0.08 to 0.23)
**Ethnicity**	79 (77)				
White—*British, Irish, any other white background*		79.2 (75.1–83.4)	79.6 (74.1–85.1)	77.6 (68.5–86.7)	80.9 (71.5–90.4)
Mixed—*White and Black Caribbean, White and Black African, White and Asian, any other mixed background*		3.6 (2.4–4.8)	3.2 (1.8–46)	3.2 (1.2–5.3)	4.5 (0.9–8.2)
Asian or Asian British—*Indian, Pakistani, Bangladeshi, Any other Asian background*		7.4 (5.5–9.3)	7.9 (4.8–10.9)	7.1 (4.2–10.1)	6.4 (3.1–9.7)
Black or Black British—*Caribbean, African, Any other Black background*		4.1 (2.9–5.2)	4.1 (2.4–5.9)	4.0 (1.8–6.1)	3.8 (1.5–6.0)
Chinese		0.3 (0.07–0.5)	0.3 (0.007–0.5)	0.2 (−0.2 to 0.7)	0.3 (−0.04 to 0.7)
Other ethnic groups		3.5 (2.5–7.3)	4.9 (2.5–7.3)	2.9 (0.6–5.1)	0.7 (0.001–1.4)
**Age**	79 (77)				
<18		0.3 (0.2–0.4)	0.3 (0.1–0.6)	0.2 (0.05–0.4)	0.2 (−0.01 to 0.5)
18–19		1.2 (0.8–1.6)	0.9 (0.5–1.3)	1.5 (0.6–2.5)	1.5 (0.7–2.3)
20–24		3.7 (3.0–4.4)	3.9 (2.9–4.9)	3.0 (1.7–4.4)	4.2 (2.8–5.5)
25–34		9.4 (8.2–10.6)	8.2 (6.7–9.7)	10.3 (7.8–12.7)	12.2 (9.9–14.6)
35–44		19.4 (17.1–21.6)	19.9 (16.4–23.3)	17.5 (13.5–21.6)	19.5 (15.8–23.3)
45–54		27.8 (25.0–30.5)	26.7 (23.2–30.3)	28.5 (23.0034.0)	29.8 (21.4–38.1)
55–64		21.6 (19.4–23.8)	22.4 (19.5–23.4)	23.4 (18.5–28.3)	16.8 (13.2–20.4)
65–74		11.8 (10.2–13.4)	12.9 (10.3–15.4)	10.4 (7.9–13.0)	10.7 (7.8–13.6)
75+		4.8 (4.0–5.6)	4.7 (3.4–5.9)	5.0 (3.7–6.3)	5.0 (3.0–7.0)
**New to the service**	79 (77)				
Patient new to service		54.2 (49.7–58.7)	55.7 (49.9–61.7)	51.7 (42.8–60.5)	52.7 (39.3–66.1)
Patient previously seen by service		44.5 (40.1–49.0)	44.2 (38.3–50.1)	48.3 (39.5–57.2)	39.6 (27.5–51.7)
**Drinking risk level**	78 (76)				
Hazardous drinking^b^		12.6 (9.7–15.4)	13.2 (9.4–16.9)	8.5 (4.8–12.1)	17.5 (7.8–27.1)
Harmful drinking^c^		17.0 (14.8–19.2)	15.8 (12.8–18.7)	19.2 (14.5–23.9)	17.7 (12.7–22.7)
Moderate dependence^d^		28.8 (25.5–32.0)	27.1 (22.9–31.3)	29.5 (22.8–36.2)	32.7 (24.8–40.6)
Severe dependence^e^		66.2 (36.8–95.7)	71.0 (17.9–124.1)	76.6 (51.0–102.1)	38.6 (25.9–51.3)
**Alcohol-related liver disease (ArLD)**	78 (76)				
ArLD—all *Of which:*	77 (75)	32.9 (28.6–37.3)	33.6 (27.5–39.7)	37.4 (28.5–46.2)	25.1 (16.3–33.9)
ArLD—compensated		56.3 (50.9–61.7)	59.4 (52.3–66.5)	50.2 (39.8–60.7)	52.3 (38.4–66.2)
ArLD—decompensated		38.1 (33.2–43.0)	40.6 (33.5–47.7)	36.0 (27.3–44.6)	36.9 (25.2–48.7)
**Alcohol-related brain damage (ARBD)**	77 (75)	13.9 (11.3–16.4)	16.2 (12.7–19.7)	14.3 (9.0–19.7)	6.6 (3.4–9.8)
**Coexisting severe mental illness**	78 (76)	14.1 (11.2–16.9)	14.9 (11.2–18.6)	17.2 (10.5–23.9)	6.8 (3.9–9.6)
**Coexisting common mental disorders**	77 (75)	62.2 (57.3–67.1)	58.2 (52.2–64.2)	69.3 (59.4–79.2)	63.1 (47.7–78.5)
**Coexisting substance use**	77 (75)	25.6 (21.5–29.8)	21.2 (17.0–25.4)	35.2 (25.1–45.4)	26.5 (15.3–37.7)

(*Continued*)

**Table 2:** Continued

			Hospital size (based on no. of discharges per month) (*n* = 77)		
	Total *n* full sample (by hospital size)^a^	All ACTs mean % (95% CI)	Small (500–1999) mean % (95% CI)	Medium (2000–2999) mean % (95% CI)	Large (3000+) mean % (95% CI)
**Experiencing homelessness**	79 (77)	14.3 (11.6–17.0)	13.8 (10.3–17.4)	19.8 (13.6–25.9)	8.0 (5.0–11.0)
**Frequent attenders to ED (3+ in last 6 months)**	78 (76)	27.5 (23.2–36.0)	29.8 (23.6–36.0)	29.3 (21.7–36.9)	13.4 (10.7–16.2)
**Frequent attenders admitted (3+ in last 6 months)**	78 (76)	21.2 (17.0–25.5)	22.9 (16.7–29.0)	23.3 (14.5–32.2)	11.8 (8.0–15.5)
**Mean number of referrals per month**	80 (77)	146 (126–167)^a^	121 (98–144)^a^	191 (152–229)	169 (99–238)
**Mean number of assessments per month**	80 (77)	138 (117–159)^a^	114 (89–139)^a^	182 (141–223)	158 (93–223)
Mean % of referrals seen		95	94	95	93
**Referral sources (mean % referrals)**	80 (77)				
Emergency department		43.6 (38.5–48.6)	44.5 (37.3–51.7)	47.5 (37.5–57.5)	33.2 (22.2–44.3)
Inpatient (admitted)		50.0 (44.6–55.4)	48.5 (40.7–56.3)	49.0 (38.6–59.4)	57.0 (44.1–69.9)
Intensive care		2.9 (2.2–3.7)	3.3 (2.2–4.4)	2.0 (0.9–3.0)	3.0 (1.7–4.3)
Hospital outpatients		3.1 (2.1–4.0)	3.7 (3.0–5.2)	1.6 (0.5–2.7)	4.0 (2.2–5.8)
**Methods of referral (mean % referrals)**	80 (77)				
Generated by service		33.5 (26.3–40.6)	30.8 (20.8–40.8)	32.6 (18.8–46.4)	46.0 (28.4–63.6)
Telephone		15.2 (11.1–19.2)	17.4 (11.1–23.7)	11.2 (5.2–17.2)	10.8 (4.4–17.1)
Email/electronic referral		51.4 (43.7–59.1)	51.8 (41.0–62.7)	56.2 (41.0–71.4)	43.2 (26.5–60.0)

^a^Three hospitals with ACTs did not complete the question about hospital size and data on number of discharges per month for these hospitals were not publicly available. This column shows how many hospitals with ACTs responded to the question overall and (in brackets) how many responded to this question and also gave their hospital size. The number in brackets is therefore the total sample per item for the breakdown by hospital size.

^b^‘Drinking above 14 per units week or AUDIT Score 8–15’.

^c^‘Experiencing social, psychological or physical problems due to drinking but NOT dependence, or AUDIT Score 16–19’.

^d^‘Experiencing significant level of tolerance, alcohol withdrawal, impaired control, typically may be drinking 15–30 units per day’.

^e^‘Experiencing severe alcohol withdrawal, high tolerance, typically drinking 30 units or more per day and may have experienced withdrawal seizures or delirium tremens’.

Just over half of patients seen were new to the ACT service compared with those already known to the service and most patients seen by ACTs experienced severe alcohol dependence (see Table 2).

Approximately one-third of patients seen by ACTs had ArLD and ~14% patients additionally presented with alcohol-related brain damage (ARBD), coexisting severe mental illness, and/or experiencing homelessness.

Just over a quarter of assessments involved frequent attenders to ED (three or more attendances in the past 6 months) and just over one fifth were with patients admitted frequently as inpatients (three or more admissions in the past 6 months).

#### Referrals

The mean number of referrals to ACTs was 146 per month (95% CI 126–167) (see Table 2). The smallest hospitals received the fewest referrals per month, whilst the medium sized hospitals received the most referrals per month (see Table 2).

Most referrals to ACTs were received from inpatient wards followed by referrals from ED. Just over half of referrals received by ACTs came via electronic patient record and email, whilst a third of ACT patients were identified directly by the team.

### Aim 3: To identify the service structure and components of care offered by alcohol care teams in England.

#### Leadership, staffing, and operating hours (see Table 3)

**Table 3:** Structure and components of alcohol care teams in England

STRUCTURE	Total *n* full sample (by hospital size)	*N* (%)	Hospital size (based on no. of discharges per month) (*n* = 77)		
		Mean (all ACTs) (95%CI)	Small (500–1999)	Medium (2000–2999)	Large (3000+)
**Hospital size**	77	77	43 (54)	21 (26)	13 (16)
**Clinical leadership**					
Identified clinical lead? Yes	80 (77)	74 (93)	37 (86)	21 (100)	13 (100)
Min. 0.2 WTE clinical lead? Yes	80 (77)	46 (58)	26 (60)	8 (38)	11 (85)
Clinical lead specialty:	74 (71)				
Addiction		19 (25)	11 (30)	3 (14)	4 (31)
Gastro/hepatology		39 (53)	20 (54)	13 (62)	5 (38)
Psychiatric liaison		2 (3)	1 (3)	0	1 (8)
Emergency medicine		6 (8)	2 (5)	1 (5)	2 (15)
Other		8 (11)	3 (8)	4 (19)	1 (8)
**Mean WTE of clinical lead (doctor)**	46 (45)	0.2 (0.1–0.3)	0.2 (0.1–0.3)	0.2 (0.04–0.3)	0.4 (0.2–0.7)
**Mean WTE of clinical lead (nurse)**	22 (21)	0.7 (0.6–0.9)	0.7 (0.5–0.9)	0.8 (0.4–1.2)	0.6 (−0.2 to 1.4)
**Mean number of clinical staff**	80 (77)	3.6 (3.1–4.0)	3.1 (2.4–3.7)	3.8 (2.9–4.7)	4.8 (3.9–5.7)
**Operating hours**	80 (77)				
<5 days/wk		0	0	0	0
5–6 days/wk		33 (41)	22 (51)	8 (38)	1 (8)
7 days/wk		47 (59)	21 (49)	13 (62)	12 (92)
Total hours operational/wk (mean)		58.6 (55.1–62.1)	57.1 (52.0–62.2)	55.3 (49.6–61.0)	70.5 (64.8–76.1)
**COMPONENTS**	**Total *n* full sample (by hospital size)**	**All ACTs *N* (%) mean % (95% CI)**	**Hospital size (based on no. of discharges per month) (*n* = 77)**		
			**Small (500–1999)**	**Medium (2000–2999)**	**Large (3000+)**
**Identification and brief advice**	80 (77)				
ED		63 (79)	33 (77)	19 (90)	9 (69)
Inpatient wards		77 (96)	41 (95)	20 (95)	13 (100)
Provides training?		74 (93)	39 (91)	20 (95)	12 (92)
**Medically assisted alcohol withdrawal (MAAW)**	78 (75)				
Number per month (mean)		75.6 (61.5–89.8)^a^	66.1 (47.1–85.1)^a^	105.7 (73.4–137.9)	63.1 (48.9–77.3)
Number per month (categorical)					
*0*		1 (1)	1 (2)	0	0
*1–49*		29 (38)	21 (50)	3 (14)	3 (25)
*50–99*		32 (41)	13 (31)	11 (52)	8 (67)
*≥ 100+*		16 (21)^a^	7 (17)^a^	7 (33)	1 (8)
Average length of stay (days)		4.1 (3.7–4.4)	4.1 (3.6–4.6)	3.9 (3.3–4.5)	4.4 (3.8–5.0)
Medication strategy					
*Fixed dose (mean % assessments)*		41.0 (32.8–49.3)	45.1 (33.6–56.7)	25.1 (11.4–38.8)	57.9 (37.6–78.2)
*Symptom triggered (mean % assessments)*		52.9 (44.7–61.2)	49.8 (38.3–61.3)	69.6 (55.2–84.0)	38.4 (19.1–57.7)
*Front loading (mean % assessments)*		3.4 (1.8–5.1)	2.7 (0.9–4.5)	5.3 (1.2–9.4)	3.7 (−1.4 to 8.7)

(*Continued*)

**Table 3:** Continued

COMPONENTS	Total *n* full sample (by hospital size)	All ACTs *N* (%) mean % (95% CI)	Hospital size (based on no. of discharges per month) (*n* = 77)		
			Small (500–1999)	Medium (2000–2999)	Large (3000+)
Average no. interactions per MAW		4.2 (3.7–4.6)	4.0 (3.5–4.6)	4.4 (3.5–5.3)	4.3 (2.6–6.0)
Outcome of MAW:					
*Mean % of MAW assessments commenced and completed in hospital*		59.6 (53.6–65.6)	58.4 (49.6–67.2)	65.0 (55.6–74.4)	58.7 (45.0–72.3)
*Mean % of MAW assessments commenced and did not complete or transfer for completion*		25.8 (20.8–30.8)	26.4 (19.8–33.1)	26.2 (17.2–35.2)	23.7 (9.0–38.5)
**Manualized structured interventions offered?**	80 (77)	48 (60)	23 (53)	13 (62)	10 (77)
**Mutual aid and peer support**	80 (77)				
Access to AA meetings/sponsor during stay		30 (37.5)	12 (28)	8 (38)	7 (54)
Access to SMART recovery		9 (11)	5 (12)	3 (14)	1 (8)
Access to peer mentors or people with lived experience during stay		28 (35)	13 (30)	9 (43)	5 (38)
**Initiation of relapse prevention medication**					
Offered?	80 (77)	54 (68)	28 (65)	13 (62)	11 (85)
Mean no. receiving RP medications (/month)	54 (54)	20.9 (7.5–34.3)^a^	26.5 (1.5–51.5)^a^	19.7 (7.6–31.8)	9.5 (0.9–18.0)
Of those receiving RP medications, what proportion receive acamprosate	54 (54)	91.4 (86.0–96.8)	90.9 (84.7–97.0)	94.6 (83.8–105.4)	89.2 (71.2–107.2)
**Referrals and liaison (mean % of assessments)**	80 (77)				
Sign post to single point of access or drop-in service		44.6 (36.1–53.1)	43.6 (31.4–55.9)	62.9 (48.1–77.6)	23.3 (8.6–38.0)
New referral to community alcohol team		30.4 (25.8–35.0)	29.4 (22.6–36.1)	30.4 (23.6–37.3)	32.3 (19.7–44.9)
Re-engagement with community alcohol team		26.9 (21.8–32.0)	22.7 (16.1–29.2)	38.5 (27.0–50.0)	20.2 (10.9–29.4)
Referral to alcohol inpatient service		6.4 (3.1–9.6)	7.5 (1.9–13.1)	7.5 (3.0–12.1)	1.4 (0.2–2.6)
Referral to GP		19.9 (12.2–27.6)	21.3 (10.1–32.5)	15.7 (2.9–28.5)	24.2 (3.7–44.6)
Referral to mental health team		21.6 (17.0–26.2)	23.7 (16.8–30.7)	21.2 (13.7–28.7)	10.8 (5.6–16.0)
Referral to social services		15.8 (12.2–19.6)	15.8 (11.2–20.3)	22.3 (12.7–31.8)	6.3 (3.4–9.2)
**Policies and pathways**	80 (77)				
Management of MAW		78 (98)	42 (98)	21 (100)	12 (92)
Prescribing relapse prevention medication		52 (65)	29 (67)	13 (61.9)	10 (77)
Alcohol-related liver disease care pathway		68 (85)	35 (81)	19 (90)	12 (92)
Alcohol-related brain damage care pathway		44 (55)	24 (56)	14 (67)	6 (46)

^a^One site reported a high number of referrals/assessments that may have also included community treatment activity. Inpatient versus community could not be disaggregated.

The majority of ACTs (93%) identified a designated clinical lead, though only 58% reported that this role was funded. Most clinical leads were gastroenterology/hepatology specialists (53%), followed by addiction specialists (25%), and most were doctors (62%).

All ACTs operated on a minimum of 5 days per week, with 47 services (59%) operating on 7 days per week, with mean operating hours across all ACTs of 58.6 (95% CI 55.1–62.1) h. The mean whole time equivalent (WTE) (1.0 WTE equals one full-time member of staff) of clinical staff (including nurses, health care assistants, and drug and alcohol practitioners) was 3.6 WTE (95% CI 3.1–4.0) per ACT.

#### Components of care

Nearly all (96%) ACTs reported the provision of IBA for alcohol on inpatient wards with 79% of ACTs also reporting IBA in the ED.

ACTs had an average of 76 (95% CI 62–90) assessments per month requiring a medically assisted alcohol withdrawal (MAAW) and reported the average length of stay for MAAW being 4.1 (95% CI 3.7–4.4) days. The reported length of stay was consistent across hospitals of all sizes with an ACT. Symptom-triggered MAAW medication strategies were most common in ACTs, followed by fixed dose strategies. Over half of patients started on MAAW were reportedly completed in hospital; however, one quarter were discharged prior to completion.

Sixty percent of ACTs reported delivering psychosocial interventions, more likely in larger (77%) than in smaller (53%) hospitals.

Just over one-third of ACTs reported that patients had access to alcoholics anonymous (AA) meetings or AA sponsors during their stay (37.5%) or had access to peer mentors or people with lived experience (35%). A smaller proportion had access to SMART Recovery services (11%).

More than two thirds of ACTs (68%) offered relapse prevention medication as part of their service, and the most common relapse prevention medication prescribed was acamprosate.

Just under half of assessments resulted in sign-posting patients to a drop-in or single point of access alcohol service on discharge. Just under a third of assessments resulted in a new referral to community alcohol services and just over a quarter resulted in supported re-engagement with community alcohol services. On average, less than a quarter of assessments led to referrals to general practice, mental health services, or social services.

Most ACTs had policies in place for the management of MAAW (98%) and ArLD (85%), with fewer services having policies in place for prescribing relapse prevention medication (65%) and ARBD (55%).

## Discussion

This study aimed to survey the provision of alcohol care in England and the characteristics of patients treated. Eighty ACTs were identified with an additional five that did not complete a survey. In line with the additional funding provision, the ACTs largely mapped onto the geographical locations with the highest community prevalence of alcohol dependence.

Consistent with the core service descriptor (NHSE 2019c), nearly all ACTs identified a ‘clinical lead’, although in a large minority of cases (42%) this role was not funded and could include relatively junior clinical staff. This implementation of ‘clinical leadership’ falls far short of the recommendation on which the ACT model was built (Moriarty 2011), which recommended consultant leadership with clinical input in addition to providing strategic vision for these services to be sustainable within the hospital and across the health system. Research from the USA has highlighted the need for leaders of hospital-based alcohol teams to be creative, tenacious, and adept at navigating hospital systems and funding to ensure that hospital care improves quality of care and is sustainable (Williams et al. 2025).

Nearly all ACTs reported having IBA in place across the hospital and most provided education and training to hospital staff on IBA; however, the mean proportion of referrals generated by email or electronic referral was only 51%, indicating that systematic screening and case identification were not, in most cases, being undertaken at the organizational level. Free text responses indicated that routine systematic screening was uncommon, ‘except by the ACT’ which also highlights the importance of strategic operational leadership to facilitate implementation of interventions at an organizational level. Notably, ACTs in larger hospitals attracted referrals from a wider range of inpatient risk groups and relatively fewer from ED.

Nearly all ACTs had a policy in place regarding the management of MAAW and all but one supported MAAW. The average length of stay for MAAW was 4 days which is consistent with hospital level data (Phillips et al. 2025). Data from the full survey (available on request) indicate the length of stay is longer than at sites with in-reach only services, potentially demonstrating the positive impact of ACTs on quality of care. However, acute alcohol withdrawal symptoms often persist for 5–10 days (Haber et al. 2009, Holleck et al. 2019, Alvanzo et al. 2020), suggesting that people are potentially being discharged whilst still experiencing alcohol withdrawal. Whilst it is hard to ascertain the quality of MAAW being provided from this survey, a quarter of patients commencing MAAW were discharged before completing the prescribing regime, with no plan for it to be completed elsewhere. Furthermore, a high proportion of MAAW regimes were symptom-triggered, yet the average number of interactions from the ACT staff during MAAW was 4, indicating that ward staff were largely left to manage this process. Recent evidence suggests that in more severe cases, MAAW using a symptom-triggered regime may be suboptimal in the acute hospital setting (Holleck et al. 2019, Kast et al. 2025).

More than half of ACTs reported providing some form of psychosocial intervention, though it was not possible from the data to ascertain the structure or quality of these interventions and evidence suggests that most psychosocial interventions delivered within treatment services are not delivered in line with the guidance (Brennan et al. 2016).

Pivotal to the core descriptor for ACTs (NHSE 2019c) is planning safe discharge and transition to community services, including onward referral and prescription of relapse prevention medication, which has been shown to be effective in reducing hospital readmissions (Wei et al. 2015). Despite an average of one-third of assessments reportedly resulting in a new referral to alcohol treatment, national data indicate the proportion of referrals into specialist alcohol treatment received from hospitals only increased from 5% to 8% between 2018 and 2024 (OHID 2025b). However, on average, just under half of patients seen by the ACT were still signposted to self-refer to alcohol services. Rapid access to specialist treatment is known to be associated with better outcomes (Srivastava et al. 2021) and for many people, being seen by a hospital-based alcohol team will be a first teachable/reachable moment (Williams et al. 2025). Therefore, improving liaison between hospitals and community services and rapid access to specialist services is a priority.

Nearly half of patients seen by ACTs were already known to them and a substantial proportion of patients experienced complex coexisting conditions. People with alcohol-related admissions experience high-levels of comorbidity, which impacts on treatment planning, prognosis, and patient outcomes (Phillips et al. 2025). These presentations pose a significant clinical challenge for a workforce drawn from various nursing and medical professions who need skills in the management of AUD, co-occurring medical complications, possible cognitive impairment, and safeguarding issues. Concerns have been raised about a reduced specialist workforce and staff competencies (Moriarty 2020), which have been partially addressed through the development of clinical competencies for hospital staff (Phillips et al. 2020). Continued investment in training and development of specialist staff is required to support improved patient outcomes in line with recognized quality standards (NICE 2023, Parker et al. 2023).

Consistent with evidence suggesting that 20% of alcohol-dependent patients are readmitted within 30 days (Phillips et al. 2025), around a quarter of assessments involved frequent hospital attenders. This further highlights the need for alcohol-dependent patients to remain in hospital for sufficient time to complete treatment and the need for ACT and community services to ensure that patients receive the input they need outside the hospital setting, including specialist intensive provision, such as alcohol assertive outreach treatment, for patients with complex needs who struggle to access traditional treatment services (Drummond et al. 2017, Fincham-Campbell et al. 2017, Blackwood et al. 2020).

### Conclusion

ACTs have been established across England to support alcohol-dependent patients. Systems are in place to support identification, management of MAAW and liaison with community services; however, variations in investment in clinical leadership and staff training may hamper systematic implementation of key components of care such as routine mandatory alcohol screening and case identification, optimal management of alcohol withdrawal and pathways to community specialist services.

### Limitations

All data were self-reported best estimates based on an average month in the last 12 months. There is the possibility that data may be biased according to the respondent’s range of knowledge and experience. We aimed to complete the survey with a member of the ACT with a good knowledge of the service, its functions, and its patient group to ensure the most accurate data possible.

Data on the provision of psychosocial interventions were limited by the lack of detail on the type of provision or on the inclusion of combined pharmacological and psychosocial interventions, which have been shown to be more effective than psychosocial interventions alone (Minozzi et al. 2025).

Due to funding changes, the ACT field is constantly changing. However, the authors consider it important to have a record of the situation whilst the Long Term Plan funding was still in place to draw future comparisons where funding has been lost or withdrawn.

## Data Availability

Data available on request. The data underlying this article will be shared on reasonable request to the corresponding author.
